# Research Progress in Skin Aging and Immunity

**DOI:** 10.3390/ijms25074101

**Published:** 2024-04-07

**Authors:** Xin He, Xinyu Gao, Weidong Xie

**Affiliations:** 1 State Key Laboratory of Chemical Oncogenomics, Shenzhen International Graduate School, Tsinghua University, Shenzhen 518055, China; hexin22@mails.tsinghua.edu.cn (X.H.); gao-xy23@mails.tsinghua.edu.cn (X.G.); 2 Open FIESTA Center, Shenzhen International Graduate School, Tsinghua University, Shenzhen 518055, China; 3 Shenzhen Key Laboratory of Health Science and Technology, Institute of Biopharmaceutical and Health, Tsinghua University, Shenzhen 518055, China

**Keywords:** skin aging, immune cells, immune function, inflammatory response

## Abstract

Skin aging is a complex process involving structural and functional changes and is characterized by a decrease in collagen content, reduced skin thickness, dryness, and the formation of wrinkles. This process is underpinned by multiple mechanisms including the free radical theory, inflammation theory, photoaging theory, and metabolic theory. The skin immune system, an indispensable part of the body’s defense mechanism, comprises macrophages, lymphocytes, dendritic cells, and mast cells. These cells play a pivotal role in maintaining skin homeostasis and responding to injury or infection. As age advances, along with various internal and external environmental stimuli, skin immune cells may undergo senescence or accelerated aging, characterized by reduced cell division capability, increased mortality, changes in gene expression patterns and signaling pathways, and altered immune cell functions. These changes collectively impact the overall function of the immune system. This review summarizes the relationship between skin aging and immunity and explores the characteristics of skin aging, the composition and function of the skin immune system, the aging of immune cells, and the effects of these cells on immune function and skin aging. Immune dysfunction plays a significant role in skin aging, suggesting that immunoregulation may become one of the important strategies for the prevention and treatment of skin aging.

## 1. Skin Aging and Immunity

### 1.1. Characteristics of Skin Aging

Aging is a complex process that affects various organs and systems of the body, including the skin [1,2]. The hallmark features of skin aging are structural and functional changes that lead to age-related skin alterations, fragility, and even disease occurrence [3,4]. Visible signs of skin aging include a reduction in collagen content, decreased skin thickness, dryness, and wrinkles [5]. Skin aging involves multiple cellular and molecular mechanisms including the free radical theory, inflammation theory, photoaging theory, and metabolic theory [5,6]. Recent research has revealed a close relationship between skin aging and disturbances in the skin immune system.

### 1.2. Introduction to the Skin Immune System and Its Functions

The skin immune system is a complex and integral part of our body’s defense mechanism [7]. It consists of various cell types, including macrophages, lymphocytes, granulocytes, dendritic cells, and mast cells, each playing a specific role in maintaining skin homeostasis and responding to injury or infection [6].

Macrophages, constituting one of the most abundant immune cell types in the skin, can be categorized into resident macrophages and those derived from blood monocytes [8]. These cells can be anti-inflammatory or pro-inflammatory. Skin macrophages are key cells in skin homeostasis and host defense that are involved in regulating chronic inflammation [9,10,11]. Dermal macrophages, as innate immune cells, play a crucial role in the dermal tissue structure and are also important for the maintenance of immune function [12].

The skin is a host for resident T cells and also recruits circulating T cells. Epidermal T cells are primarily epidermis-specific T cells, including cells such as memory T cells [13,14], playing a key role in monitoring skin pathogens and early immune responses [15]. Dermis T cells are mainly circulating T cells that migrate to the area during skin inflammation or infection [16], participating in regulating immune responses including by combating pathogens and contributing to skin inflammatory processes. Different T cell subtypes (such as Th1, Th2, Th17, and Th22) are associated with specific cytokines and effector functions [10] The skin immune system is critical for maintaining skin homeostasis and effective wound healing, playing an important role in skin aging.

Natural Killer (NK) cells in the skin are cytotoxic lymphocytes capable of destroying virus-infected cells and cancer cells without the need for antigen presentation or initiation [17]. NK cells are activated by interferons or other cytokines released by macrophages, inducing apoptosis through the perforin–granzyme pathway. Granulocytes, including neutrophils, eosinophils, and basophils, play various roles in the immune response.

Neutrophils, constituting the most abundant type of white blood cell in the body, are particularly important for combating bacterial infections and some fungal infections. In skin immune responses, neutrophils can rapidly respond to infection or injury, being among the first immune cells to arrive at the infection site [18,19].Eosinophils, associated with parasitic infections and atopic dermatitis, promote innate and adaptive immune responses by releasing cytotoxic granules and promoting T cell differentiation. They are present in the skin, especially near blood vessels, to help combat parasites and other invaders [20,21]. Basophils enter the skin in conditions such as parasitic infections and atopic dermatitis, participating in the immune response by releasing cytotoxic granules and promoting T cell differentiation.

Dendritic cells are key antigen-presenting cells that play roles in both innate and adaptive immune responses. They recognize antigens and present them to T cells. Langerhans cells, with dendrite-like projections, monitor the environment and bind pathogens to their Toll-like receptors, travel to lymph nodes, and present antigens to naïve lymphocytes. Epidermal dendritic cells (such as Langerhans cells) and dermal dendritic cells participate in innate and adaptive immune responses.

Mast cells, widely distributed in skin tissues, are activated upon contact with allergens [22], leading to degranulation and the release of inflammatory mediators like histamine, resulting in urticaria-like changes such as itching and swelling [23,24,25,26].

The skin’s various immune cells not only influence each other but also interact with keratinocytes, fibroblasts, vascular cells, and neural cells within the skin tissue, playing a positive role in immune-barrier function. The skin also hosts its own microbial community [27,28,29], which helps shape the skin’s immune function through ongoing signaling interactions with skin immune cells [30]. These commensal microbes can produce antimicrobial peptides (AMPs) and induce the expression of AMPs by host epithelial cells [29]. Commensal bacteria can also induce keratinocytes and sebaceous gland cells to produce homeostatic cytokines, expand the pool of skin CD4+ and CD8+ T cells, and stimulate skin T cells to produce cytokines [31,32].

The skin barrier can protect the immune function of the skin, and it is also crucial for skin health and aging. Skin aging includes changes in the structure of the stratum corneum and lipid content, which have a negative impact on the skin’s barrier function and lead to reduced water retention and increased vulnerability to infection [28]. The stratum corneum of aging skin becomes thinner, leading to aging.

Thus, the skin is not merely a physical barrier but possesses a complex immune system composed of various immune cells that interact not only with cells within the skin tissue but also with the skin’s microbial community [33], together maintaining the health and functionality of the skin. In this way, the skin effectively defends against the invasion of external pathogens while maintaining its own healthy homeostasis [34,35,36].

The skin is a crucial immune and neuroendocrine system, harboring microorganisms that maintain balance. Microbial transfer occurs with age and may accelerate age-related skin changes. Microorganisms play a crucial role in the human body, protecting the host, regulating metabolism, and shaping immunity. The skin microbiota maintains the epidermal barrier and resists pathogens. The efficacious restoration of the skin barrier and dysbiosis with the strategic integration of acidic cleansers, emollients with optimal lipid composition, antioxidants, and judicious photoprotection may be a proactive approach to aging. Furthermore, the modulation of the gut–skin axis through probiotics, prebiotics, and postbiotics emerges as a promising avenue to enhance skin health as studies have substantiated their efficacy in enhancing hydration, reducing the number of wrinkles, and fortifying barrier integrity [28].

### 1.3. Aging of the Skin Immune System and Defensive-Functional Changes

Current research indicates that it is generally believed that endogenous factors such as genetics, as well as external environmental factors such as ultraviolet radiation, wind exposure, and smoking, can affect skin aging. In addition, the immune capacity of the skin can also affect the degree of skin aging. The following discussion mainly focuses on the immune functional change aspect of skin aging.

With the extension of life spans, the skin immune system undergoes aging [37,38,39], leading to corresponding defensive-functional changes. When the immune system ages, not only does the self-renewal capability of immune cells decrease but their functions also change, resulting in an overall decline in immune defensive function. Because immune cells have a certain capacity to clear aging cells [40], the decline in immune function affects the clearance of aging skin cells, influencing the occurrence of skin aging. The decline in immune function also leads to decreased responsiveness to pathogens, promoting the incidence of skin diseases.

As the skin immune system ages, the functions of many types of immune cells change, not only impacting skin aging itself but also significantly reducing skin’s defensive functions. The autophagic capacity of immune cells in the skin decreases, leading to a diminished ability to clear aging cells from the tissue. The cytotoxic action of T cells against aging cells and the phagocytic capability of macrophages towards aging cells both decline, causing aging cells to linger for extended periods [41,42]. Therefore, it is evident that the decline in immune cell function impacts skin aging. The number and function of memory T cells also change during aging, possibly leading to a reduced response capability to specific antigens. The secretion of TNF-α by macrophages decreases, affecting the immune system’s ability to effectively detect and respond to pathogens. The number of regulatory T cells (Tregs) increases in aging skin, potentially leading to the reactivation of infectious diseases or altering the inflammatory microenvironment by suppressing Th1 and Th2 response [43].

The increase in regulatory T cells in aging skin might also be related to the weakened antigen-specific T cell responses. Aging is associated with a reduction in the skin immune surveillance function of memory CD4+ T cells. The decline in immune surveillance is partly due to the reduced synthesis of TNF- α by macrophages, affecting the immune system’s ability to effectively detect pathogens and respond to them [44]. The chemotactic ability of neutrophils and the phagocytic capability of macrophages are impaired, affecting their ability to regulate inflammation and resist microbial infections [45]. Immune cells experience metabolic disorders affecting their energy production and utilization and thereby impacting their activation and proliferation capabilities. The migration ability of aging Langerhans cells (LCs) decreases, affecting their response to harmful stimuli in the epidermis and their detection of pathogens. Their production of antimicrobial peptides (hBD3) decreases, affecting defense against infections. In the skin, dendritic cells (DCs) are primarily responsible for capturing, processing, and presenting antigens to activate T cells. With aging, dendritic cells undergo functional decline, affecting the skin’s immune response to pathogens and tumor cells. Aging dendritic cells may reduce the secretion of key cytokines IL and TNF, thereby affecting their ability to activate T cells [46].

When immune cells age, functional changes involve many cellular and molecular mechanisms. The genomes of immune cells become less stable, with telomere shortening, reduced cell division capability, and increased mortality. The division and proliferation capability of aging immune cells weaken, slowing down the renewal rate of immune cells and affecting the overall function of the immune system [47]. The occurrence of apoptosis increases during aging, especially in aging T cells, due to the decreased expression of anti-apoptotic proteins (such as Bcl-2), increasing the tendency towards apoptosis [48].

Changes in external factors can trigger cellular aging and inflammatory responses in the skin. Chronic low-grade inflammatory stimuli counteract immunosuppression, in-volving through the expansion of immunosuppressive cells. Immunosuppressive activity not only inhibits the function of effector immune cells but also increases the anti-inflammatory and immunosuppressive activities of immune aging. The molecular mechanism of the response suggests that inflammation is related to the increased anti-inflammatory and immunosuppressive activities that promote immune aging [49].

As immune cells age, changes occur in gene expression patterns and cell division decreases, leading to a decline in immune response functions [50,51]. In aging immune cells, the expression of some key regulatory genes may change, with an increased activity of apoptosis-related genes like p53 and decreased expression of genes promoting cell survival and proliferation like Bcl-2 [52]. These changes affect cell function and responsiveness, including cytokine production and response [53].

During immune cell aging, proteostasis is lost, and internal signaling mechanisms within immune cells also change. The T cell receptor (TCR) signaling capacity of aging T cells decreases [54], and this is associated with decreased expression levels and functionality of the key signaling molecule ZAP-70 (tyrosine kinase) [55]. Additionally, the activity of NF-κB in aging cells changes, affecting the cells’ ability to respond to immune stimuli [56].

In addition to age, many internal and external factors can accelerate the aging of the skin immune system. Internal factors include vitamin D, which is an important physiological substance. Vitamin D becomes essential for antimicrobial defense by producing cathelicidin (LL37) and modulating the phagocytic activity of macrophages and NK cells. Vitamin D3 declines in aging skin, which might not normally keep the defensive functions of skin immune cells well.

In addition to these, external factors include exposure to ultraviolet radiation (UVR), air pollution, nutritional habits, and smoking [6,57,58]. Ultraviolet radiation is the most important factor leading to skin aging, and its damage to skin aging is dose-dependent. It can be observed both in vivo and in vitro, and hydrogen peroxide can also have an impact on skin aging, as hydrogen peroxide participates in UV-induced antigen presentation inhibition. After being stimulated by ultraviolet or hydrogen peroxide, skin cells can cause damage to immune function [59].To be particular, UVR can significantly downregulate the defensive functions in immune cells. These external stresses cause oxidative damage, affecting the skin’s antioxidant system, which weakens with age, leading to the aging of skin immune cells [60] and affecting immune cells’ defensive function.

Therefore, as age increases and under the accelerated impact of internal and external environmental factors, the immune cells of the skin age, leading to defensive functional changes that not only directly affect the clearance of aging skin cells but also impact the skin’s immune-barrier function. The pathophysiological process involves a wide range of molecular mechanisms. Understanding the functional changes and molecular and cellular mechanisms of aging skin immune cells is of great significance for developing new anti-aging strategies for the skin and preventing the occurrence of skin-related diseases [61,62].

### 1.4. Effects of Inflammation Caused by Hyperimmune Function on Skin Aging

Cell death may be triggered by inflammation, which is particularly related to the skin. The skin is a tissue that provides a structure and immune barrier to maintain life with the environment. Keratinocyte death is a prominent histopathological feature of many inflammatory skin diseases, and epithelial cell death may contribute to the pathogenesis of skin inflammation [63]. Indeed, inflammation plays an important role in skin cell survival and also includes cell senescence.

Inflammatory senescence is a chronic [64], low-grade, age-related inflammatory state that is caused by various factors such as oxidative stress [2,65], DNA damage [66], and cell metabolism disorder [67]. It is directly related to immune cell senescence in addition to non-immune-cell-source senescent tissue cells in the body. Aging has an impact on inflammation. At the same time, aging is also the cause of inflammation. For example, inflammation will increase the secretion of “SASP”, aging will promote the secretion of inflammatory factors, and it will also have some effects on autoimmune cells such as in the over-activation of T cells [68,69,70,71].

Two of the main manifestations of immune aging are the increase in inflammatory response and the increased expression of inflammatory factors [72], which are closely related to skin aging [73]. These inflammatory factors will act on some surrounding cells, resulting in a state of growth arrest, forming the so-called inflammatory aging [74,75]. Inflammatory aging can lead to increased oxidative stress, which can lead to cell growth arrest [76]. Improving these inflammatory responses can more effectively promote skin resistance to aging.

Studies have found that in the process of skin aging, there will be inflammatory changes in the skin tissue after aging [77]. The expression of specific immune cells, such as CD4+ helper T cells, γδT cells, and innate lymphocytes, is significantly changed in the skin. Skin immune cells such as autoreactive immune cells (Th17) will be over-activated and the ability of macrophages to self-regulate inflammation will also be impaired [78]. These will cooperate with aging skin tissue cells to induce low inflammatory response, produce a large number of reactive oxygen species and a cell-secretion-senescence-related secretion phenotype (SASP), affect the skin microenvironment, and lead to skin aging. Excessive reactive oxygen species production can lead to the imbalance of cell homeostasis and promote the process of skin aging. In particular, cytokines from the IL-17 family are increased in expression in these cells, which exacerbates the chronic inflammatory state of aging skin [79]. Blocking the activity of IL-17A and IL-17F is considered a potential strategy to delay skin aging [80,81,82].

The over-activation of skin immune cells involves a variety of intracellular inflammatory signaling pathways and molecular mechanisms that promote skin cell aging. First, aged skin cells accumulate and maintain high metabolic and secretory activity even if they are unable to divide. These senescent cells exhibit altered secretions, known as aging-related secretory phenotypes (SASPs), that significantly disrupt the skin microenvironment. SASPs can continuously promote cellular senescence or affect the surrounding tissue environment, thus affecting the entire organism. SASP factors include pro-inflammatory and immunomodulatory cytokines, chemokines, proteases, and growth factors [83]. Oxidative stress leads to the oxidative damaging of biomolecules (especially DNA), resulting in endogenous DAMP and cytokine release, activating downstream pattern recognition receptor signaling pathways, and triggering systemic chronic inflammation in vivo [84]. And this oxidative stress and inflammation can interact. Necroptosis is a mode of cell death involving inflammation, resulting in the release of cell contents including DAMPs, which further triggers inflammation. This long-term inflammation can promote the growth of skin cells to arrest, promote the aging of skin cells and even death.

In addition to the above-mentioned inflammatory mechanisms, several intracellular and extracellular environments or molecular mechanisms have been identified as key factors in skin aging. These include the release of matrix metalloproteinases (MMPs) in inflammatory conditions that destroy the structural components of skin collagen. These mechanisms can lead to changes in skin structure and function, and they also provide a range of therapeutic targets for the treatment of skin aging [85,86]. In general, the activation of skin immune cells promotes skin aging through a variety of ways, mainly including promoting the secretion of SASPs, promoting skin cell growth stagnation and aging, and skin structure and function changes. Together, these processes lead to structural and functional changes in aging skin. Understanding these mechanisms provides valuable insights for developing interventions aimed at alleviating skin aging.

Many factors can lead to the hyperfunction of skin immune cells; in addition to age, many internal and external environmental factors can affect the activity of skin immune cells [87,88]. The influence of various environmental factors on skin immune cells is a complex and multifaceted research field [89].

Skin is a key neuron–endocrine system, and neuron–endocrine systems play an important role in the regulation of immune cells in skin. Emerging studies have indicated that internal factors include some important physiological substances; e.g., vitamin D3 plays an important role in skin aging and also works via immune-regulatory mechanisms. Vitamin D3 can regulate skin’s innate and adaptive immune systems [90,91]. Vitamin D3 can induce Tregs and T helper-2 (Th2)-Lym, together with the downregulation of pro-inflammatory Th1/Th17/Th9-Lym. Vitamin D3 can suppress the production of inflammatory cytokines in immune cells and also enhances the levels of anti-inflammatory IL-10 from Tregs or Th2-derived IL-4. However, when aging, the level of vitamin D3 is decreased [92,93]. The deficiency of vitamin D3 is associated with increased inflammation in skin [57,58,94].

Melatonin is an important secretion substance in our body and exerts a wide effect. Its decrease may be associated with increased oxidative stress and activated inflammation [95,96]. In a condition of aging, melatonin preferentially exerts anti-inflammatory activities against a low-grade inflammation, which is likely mediated by stimulating SIRT1 and downregulating the TLR4/NFκB pathway [97,98].

In addition to these, UVR is the most harmful external factor responsible for photoaging in skin. UVR would activate skin immune cells and lead to the development of inflammation. Both UVA (315–400 nm) and UVB (280–315 nm) contribute to photoaging in skin likely by increasing ROS, damaging mitochondrial DNA, accumulating p53 protein, increasing the number of MMPs, and inhibiting hyaluronan synthesis [99,100]. Among these, some key inflammatory pathways, e.g., NFκB and MAPK, are activated by UVR, and downstream inflammatory factors are significantly increased [101].

Research on scientific models for assessing the effects of pollutants on the skin emphasizes that both chemical and physical pollutants can alter the response of the entire organism, leading to a variety of pathologies, including those of the skin system [102,103,104]. Contaminants can affect skin by altering skin elasticity, thickness, epidermal barrier strength structure, and dermal extracellular integrity. They also trigger an intensified skin inflammatory response and regulate several cytokines and oxidative stress responses, leading to apoptosis. The global increase in air pollution poses a major threat to human health, with air pollutants affecting the physiology of the skin and causing skin damage.

Some air pollutants are photoreactive and can be activated by ultraviolet radiation, thus enhancing their harmful effects on the skin. These pollutants can also affect vitamin D synthesis by reducing UVB radiation [105]. Air pollution can cause oxidative stress in the skin and activate inflammation. Aromatics receptors (AhR) play a critical role in the response to air pollutants [106], and their downstream regulatory pathways significantly influence skin phenotypes [107]. Each of these factors influences the immune response of the skin in a unique way, resulting in the complexity of skin immunology.

The specific cytokines and molecular mechanisms involved in these responses can be highly variable, depending on the type of stimulation and the individual’s unique skin biology. Irritations such as mites and parasites may affect the skin immune response through specific cellular and molecular mechanisms [108]. The cells involved include T cells, macrophages, dendritic cells, etc., which participate in the response by releasing factors such as interleukin and tumor necrosis factor. These factors regulate the inflammatory response of cells and the aging process through specific signaling pathways such as the NF-κB pathway. Understanding these influencing factors is critical to developing targeted treatments for various skin conditions and preventing skin damage from environmental exposure [109].

## 2. Immune Regulation Strategies for Skin Aging

### 2.1. Strategies or Related Products Aiming to Enhance Skin Immune Cell Function

Senescent skin immune cells show an overall decline in immune function, which not only affects the clearance of senescent skin cells but also increases the risk of infectious diseases or further accelerates skin aging through secondary infectious inflammation. In response to these changes, aggressive strategies and products can be employed to boost the skin’s immune function. Enhancing the immune capacity of senescent skin cells can improve the ability to clear senescent skin cells and related pathogens, constituting one of the important strategies to combat skin aging [110].

In the field of skin aging, improving the immune ability of immune cells plays a significant role in resisting skin aging [111,112]. Through the improvement of immunity or the ability to remove senescent cells in the skin, the skin’s ability to resist external microbial infection is enhanced, and the skin’s immune defense ability and health level are improved [113,114].

However, in this field, present strategies or related products are defective. A few studies have reported that vitamin D3 and its metabolites seem to have some effect on enhancing the defensive functions in immune cells [115,116]. But specific products for enhancing the defensive functions in immune cells against skin aging are lacking [57,58].

Actually, we have many methods to activate immune cells and enhance immune-defensive functions. Some internal (decreased vitamin D) or exogenous factors (increased UVR) may decrease immune cell functions, and we can solve these issues point to point [101]. We can increase vitamin D levels in skin by locally or systemically administrating vitamin D3 or related products [117]. Also, we can use sunscreen products to prevent UVR damage in skin. If those factors cannot be well controlled, we can specifically rehabilitate those immune cell functions [118]. PD-1 is an inhibition factor affecting immune function. We might be able to use some inhibitors of immunosuppressive factors, e.g., the PD-1 antibody, to activate the skin’s immune system [119]. Some microbial, cellular, or protein products are claimed to be able to reprogram our immune system. In addition to these, lots of natural products including plant extracts or components have wide activities in terms of immune rehabilitation [120].

**Figure 1:**
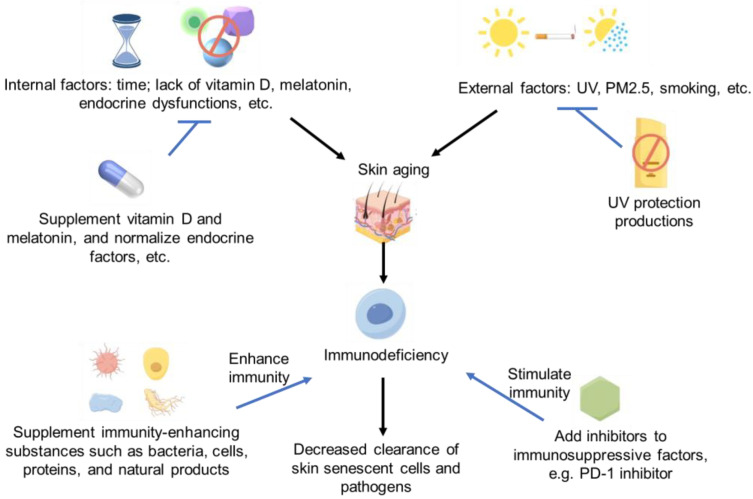
Immune deficiency and skin aging strategies Some images were sourced from Figdraw.

Therefore, in future studies, more strategies or biochemical/natural products with significant immune enhancement could be found to improve skin aging [121] by improving skin’s ability to clear aging cells or enhancing the defensive function of immune cells in skin. However, lots of investigations need to be conducted in the future. The immune regulation strategies of skin aging and immunodeficiency are summarized in Figure 1.

### 2.2. Anti-Immune Cell Over-Activation/Anti-Inflammatory Strategies or Related Products

Aging skin cells themselves are in a state of low inflammation; some immune cells are over-activated with immune hyperactivity and inflammatory aging, which have a certain impact on the overall aging of the skin. So, inhibiting immune cell over-activation or anti-inflammatory properties is one of the important strategies [122].

Since internal and external factors are important induction factors for the activation of immune cells, we should strictly regulate those internal and external factors [123]. Melatonin as an important secretion factor from the neuron–endocrine system might be decreased in aging skin and contribute to the activation of the skin’s immune system [97]. So, we might be able to use melatonin or related products locally or systemically since they can act in a local or systemic way.

UVR can directly damage skin cells and activate the immune system and cause skin inflammation and aging [101]. Some sunscreen products could be used to avoid the overaction of immune cells and prevent the damage of UVR. In addition to these, vitamin D3/melatonin and related products can directly attenuate the inflammatory damage of UVR in skin [58,97].

**Figure 2:**
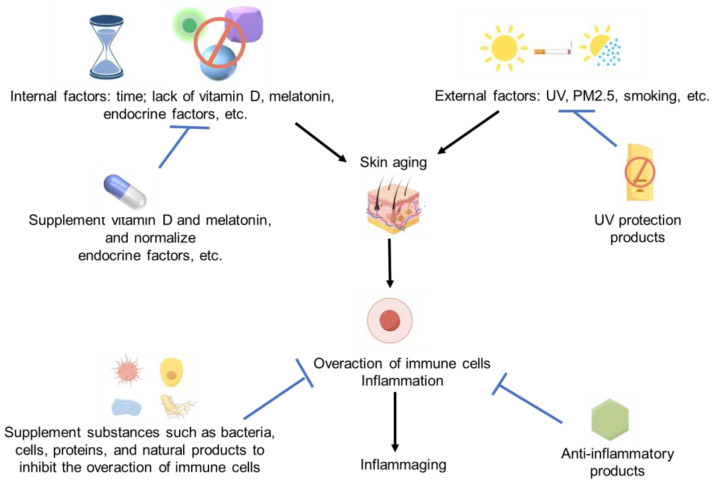
Immune activation and skin aging strategies. Some images were sourced from Figdraw.

Since the overaction of immune cells and inflammation can induce skin aging, we believe these anti-inflammatory products should have a huge potential to resist skin aging. Lots of cellular, microbial, protein-based, and natural products have shown significant anti-inflammatory activity. So, it should be not difficult for us to find some effective anti-aging products for skin [120,124]. The immune regulation strategies for skin aging and immune activation are summarized in Figure 2.

## 3. Summary and Prospects

This paper has comprehensively discussed the complex relationship between skin aging and immunity, including the characteristics of skin aging, the function of the skin immune system, the effect of the functional decline of immune aging on skin aging and function, and the effect of inflammation caused by immune hyperfunction on skin aging. The roles of various cell types (such as macrophages, dendritic cells, T cells, etc.) in skin immunity and how these cells change functionally with age or environmental stress have been described in detail. In addition, immunosenescence and its relationship with the chronic inflammatory state, oxidative stress, and gene expression patterns have also been discussed.

Skin aging is a multifaceted process that involves changes at the cellular and molecular levels such as through reduced collagen content, reduced skin thickness, dryness, and the formation of wrinkles. With an increase in age, the functions of skin immune cells such as macrophages, lymphocytes, granulocytes, and dendritic cells will undergo significant changes such as in reduced migration ability and the increased production of inflammatory factors. These changes further affect the skin’s ability to clear skin senescent cells and its defense against microbial infection, thus affecting the skin immune cells’ ability to maintain skin health. On the other hand, the hyperfunction of immune function caused by skin aging, including that of immune cells, and the low-grade inflammatory state will lead to an increase in the expression of inflammatory factors, which will aggravate the damaging of skin tissue and the occurrence of aging. In addition to age, many internal and external environmental factors will affect the function of skin immune cells and promote the occurrence of skin aging.

The most important biological processes involved in skin aging include DNA repair and stability, mitochondrial function, cell cycle and apoptosis, the extracellular matrix, lipid synthesis, ubiquitin-induced protein breakdown, and alterations in cellular metabolism [125]. In addition to these common anti-aging methods, one can also enhance the immune function of skin cells, such as by enhancing the immune clearance function of T cells in peripheral blood vessels of the skin and inhibiting the occurrence of inflammatory factors. Indeed, emerging studies have indicated that immunity also plays an important role in skin aging.

Strategies to combat skin aging involve many aspects. We can look for suitable products to enhance the function of immune cells, improve the ability to clear senescent cells, maintain the normal defense function of skin immune cells, and inhibit the occurrence of secondary inflammation. We can also look for suitable products to inhibit the over-activation of immune cells and inhibit the occurrence of inflammation and aging. Physiological active factors have anti-inflammatory activity and play a very important role in inhibiting the inflammatory aging of the skin, but the specificity and mechanism uniqueness of products are still lacking, and further research is needed. At present, there is a relative lack of products to improve skin immune function and remove skin aging cells, research is also difficult, and breakthroughs need to be made in this area in the future.

Some of the references cited in this paper are on the aspects of skin aging and immune enhancement, and reveal the potential effects on skin aging through immune function improvement. However, their limitation may be that they do not provide those strategies in more detail for skin aging resistance from an immune perspective. In future articles, we can develop more effective methods for skin aging from an immune perspective.

In future research, researchers can conduct more comprehensive experiments on skin cells and animals to construct models of immune dysfunctions and aging and then conduct drug evaluation experiments. These candidate drugs can be drugs that enhance the skin’s immune function or inhibit the overaction of skin immune cells. After regulating immune dysfunctions, the skin-aging degree can be tested, such as by detecting the activity of β- galactosidase (an important indicator of skin aging). Maybe we can build a high-through or high-content sifting method to obtain effective products by targeting the skin immune system based on advanced molecular and cellular technologies.

In this review, the complex relationship between skin aging and immunity has been discussed in depth, and relevant anti-aging strategies have been proposed from the perspective of immunology, which has enriched the connotations of the theory of skin aging. In future studies, it is necessary to better understand the cellular and molecular mechanisms of skin immune aging, and develop specific skin anti-aging products based on the relationship between skin immunity and aging, so as to develop more effective anti-aging therapies to improve skin health and improve people’s quality of life.
